# Preliminary results for 6 minute walk values in healthy German children

**DOI:** 10.1186/1546-0096-9-S1-P74

**Published:** 2011-09-14

**Authors:** Ivan Foeldvari, Günther W Himmelmann

**Affiliations:** 1 Hamburger Zentrum fuer Kinder- und Jugendrheumatologie, Germany

## Introduction

6 minute walk is a primary outcome measure in therapeutic studies for patients with pulmonary hypertension. Currently we have a two of sets of data [1,2] regarding test results in the 6 minute walk test (6MWT) in healthy children with a large span in the norm values in the different age groups.

## Aim of the study

To establish norm values for healthy German children for the 6 Minute Walk Test.

## Method

The team of an occupational therapist and a study nurse is visiting schools, were previously the parents agreed on the participation of the students on the test. Always just students from one class are invited to participate in the test. The students are performing the test according the international guidelines. The demographic data of the students are collected and the parents fill out a short survey regarding the physical activity and the health condition. Children with chronic diseases, which decrease the stamina are excluded.

## Results

Up till now 354 students participated from the age 7 to 12 years. 22 in the age group 6; 49 in the age group of 7; 61 in the age group of 8 years; 64 in the age group of 9 years ; 50 in the age group of 10 years; 51 in the age goup of 11 and 57 in the age group of 12. The mean 6 minute walk distance was 449.1 m in the age group of 6, 470 m in the age group of 7; 484 m in the age group of 8 ; 491,6 m in the age group of 9 ; 471,3 m in the age group of 10, 571 m in the age group of 11 and 502,3 m in the age group of 12 years. BMI correlated with the walked distance.

## Conclusion

Our results are in the range of the patients from the UK published by Lammers et al 1 and are in significantly lower range than in the Chinese population collected data by Li et al.2. This reflects the importance of this study to gain norm values for our patient population.
